# Research Progress on Shrimp Allergens and Allergenicity Reduction Methods

**DOI:** 10.3390/foods14050895

**Published:** 2025-03-06

**Authors:** Bingjie Chen, Hui He, Xiao Wang, Songheng Wu, Qiankun Wang, Jinglin Zhang, Yongjin Qiao, Hongru Liu

**Affiliations:** Institute of Crop Breeding and Cultivation, Shanghai Academy of Agricultural Science, Shanghai 201403, China; bingjie0204@126.com (B.C.); hh18536060094@163.com (H.H.); wangxiao.0127@163.com (X.W.); wsh_magnus@163.com (S.W.); wangqkhh@163.com (Q.W.); 2020208034@stu.njau.edu.cn (J.Z.)

**Keywords:** shrimp, allergens, allergenicity reduction, research progress

## Abstract

Shrimp are highly favored by consumers for their delicious taste and rich nutritional value. However, reports of allergic reactions caused by shrimp and its derivatives have been increasing, significantly impacting consumer health and posing a growing global food safety concern. This article introduces the structure and biochemical characteristics of major allergenic proteins in shrimp, including tropomyosin (TM), arginine kinase, sarcoplasmic calcium-binding protein, myosin light chain, troponin C, and hemocyanin. Currently, there is no effective treatment for shrimp allergies, and prevention is mainly achieved by avoiding consumption. The study of shrimp allergen sensitization reduction technology is of great significance to the development of hypoallergenic or desensitized products. The article provides a detailed overview of the effects of common processing techniques, including physical, chemical, biological, and combined methods, on the allergenicity of shrimp allergens; for instance, the binding rate to immunoglobulin E (IgE) was reduced by 73.59% after treating TM with high pressure (500 MPa) at 55 °C for 10 min and the recognition rate of TM to IgE decreased by 89.4% on average after treating TM with pepsin (30 μg/mL, pH 2) for 2 h. These techniques provide references for the development of hypoallergenic aquatic products or desensitized foods.

## 1. Introduction

Shrimp is one of the largest species in global seafood trade, with consumption primarily concentrated in China, the United States, Japan, India, and some European countries such as the United Kingdom and Germany. Customs statistics indicated that the global shrimp production in 2023 was approximately 5.6 million tons. Shrimp is rich in nutritional value as a high-protein, low-fat seafood. It is abundant in vitamins, minerals, Omega-3 fatty acids, and astaxanthin [1]. Shrimp is a good source of calcium, which contributes to healthy bones and teeth. Omega-3 fatty acids are known for their cholesterol-lowering and cardiovascular disease-preventing properties [2]. As a potent antioxidant, astaxanthin helps reduce the risk of chronic illnesses and aging. It also has benefits for cardiovascular health and enhances the body’s immune resistance [3]. The frequency of consumption and popularity of shrimp products have been steadily increasing worldwide; however, an increasing number of allergic individuals are reporting severe immune reactions [4]. Shrimp belongs to the class of Crustacea, which is one of the eight major categories of allergenic foods identified by the United Nations Food and Agriculture Organization (FAO); consequently, shrimp allergy is a serious safety hazard and even life-threatening for potentially allergic people [5].

Shrimp contains large numbers of allergenic proteins; the currently confirmed include tropomyosin (TM), arginine kinase (AK), sarcoplasmic calcium-binding protein (SCP), myosin light chain (MLC), troponin C (TnC), and hemocyanin (HC) [6]. Among these, TM accounts for up to 80% of the allergies, making it the major allergen in shrimp [7]. Several additional likely allergens have been detected in shrimp, including pyruvate kinase, filamin C, myosin heavy chain and glyceraldehyde-3-phosphate dehydrogenase [8]. Allergic reactions to shrimp are primarily IgE-mediated type I hypersensitivity reac-tions. During the sensitization phase, damage to the epithelial barrier leads to increased transmission of food protein antigens, inducing epithelial cells to secrete cytokines, which upregulate OX40 on DC ligand (OX40L) on DCs. DCs further induce the transition from initial T cells to Th2 cells, which secrete cytokines such as IL-4, IL-5, and IL-13, with IL-5 recruiting eosinophils and IL-4 and IL-13 promoting class switching of B cells to produce specific IgE (sIgE) and promoting the production of memory B cells. B cells differentiate into plasma cells after maturation and produce large amounts of antibodies to the sensitizer sIgE, which bind to high-affinity FcεRI receptors on the surfaces of mast cells and basophils, placing the organism in the sensitized state. When the sensitized organism is re-exposed to the allergen, the immune response enters the effector phase, in which the allergen-derived epitope binds to sIgE on the surface of the immune effector cells, triggering IgE cross-linking, which triggers the cellular degranulation reaction and the release of inflammatory mediators such as histamine and leukotrienes, thus causing local or systemic allergic symptoms [9].

The clinical symptoms of shrimp allergy can range from mild rash and urticaria, swelling (angioedema), gastrointestinal reactions (vomiting, diarrhea) to more severe symptoms such as gastrointestinal distress (nausea, abdominal cramping, diarrhea), respiratory issues (laryngospasm, wheezing), and ocular symptoms (conjunctivitis) [10]. Because of the complex mechanisms underlying food allergies, the most effective preventive measure at present is still to strictly avoid the consumption of allergenic ingredients in shrimp. Currently, there is no trustworthy and effective method for completely curing food allergies in clinical practice. To fully ensure the safety of individuals allergic to shrimp, in addition to necessary labeling management, researchers have conducted a series of studies on allergens and their mitigation techniques, aiming to develop hypoallergenic or desensitized shrimp products. These methods include thermal treatment, high-pressure, high-intensity ultrasound, irradiation, and a variety of chemical approaches, including Maillard reaction, enzymatic treatment, and malondialdehyde crosslinking, and biological methods, as well as combinations of these techniques. Foods undergo multiple processing steps that may alter the conformation of protein, which in turn significantly influence its digestibility, absorption kinetics, and subsequent allergenic response in the immune system; therefore, choosing an appropriate processing method is essential [11]. This report aims to offer a comprehensive and up-to-date overview of shrimp allergens with a focus on elaborating the latest processing methods and their impact on shrimp allergenicity.

## 2. Prevalence and Epidemiology of Shrimp Allergy

A questionnaire survey based on 17,280 adults aged 20–44 years from 15 countries revealed that 2.3% of allergy symptoms were associated with shrimp, making shrimp allergy a major cause of crustacean allergy [12]. In a retrospective analysis of seafood-allergic patients at three allergy clinics at the Texas Medical Center between 1 January 2010 and 30 January 2010, it was found that shellfish allergy (59.1%) was more common than fish allergy (13.8%) in patients with an average age of 50.2 years (18–81), crustaceans (82.6%) were more common than mollusks (7.2%), and shrimp (72.5%) was the most common shellfish allergy [13]. A survey of 253 children aged 1 to 18 years from the University of Campania ’Luigi Vanvitelli’ who performed an ImmunoCAPISAC from 2017 to 2021 found that 9.1% of patients were sensitized to Derp10, with 89.6% of these patients experiencing anaphylactic reactions after shrimp or shellfish ingestion [14]; furthermore, 21 patients (8.3%) experienced anaphylaxis after shrimp ingestion [15]. Many studies have shown that its prevalence is influenced by gender, age, location, and consumption level [8]. An investigation of shrimp-allergic children under the age of 18 conducted at the Allergy and Immunology Clinic of Texas Children’s Hospital over 11 years found that 68 patients were diagnosed with shrimp allergy, of which 61% were male and 39% were female [16]. Although shrimp allergy is more common in adults and older children, it can appear at any age, and individuals who are allergic to shrimp may maintain this allergy throughout their lives. Notably, compared with adults, the frequency of individual epitope recognition and the intensity of IgE binding are notably greater in children. For example, the allergen recognition rate of the TM was 94% in children and 61% in adults [17]. The prevalence of shrimp allergies also varies by region. In some European countries, Italy and France have the highest rates of shrimp allergy at 10.2% and 7%, respectively, while Australia and Iceland have relatively lower rates at 2.4% and 2.8%, respectively [18]. A large-scale study conducted across 52 cities in Asia documented that the sensitization rate for shrimp allergy was as high as 19.97% among 44,156 allergic patients [19].

## 3. Molecular Characterization of Shrimp Allergens

Table 1 summarizes the allergens and related molecular characteristics described so far.

### 3.1. TM

Hoffman et al. first isolated an allergen from shrimp, a major allergen in crustaceans, in 1981 [31]. Daul et al. reported an allergen with a molecular weight of 34 kDa from the brown shrimp (*Penaeus aztecus*), called Pen a 1, which reacted positively with serum IgE in 82% of allergic patients [20]. Shanti et al. reported a 34 kDa heat-stable protein called Pen I 1 (*Penaeus indiana*), with IgE-binding epitopes located in peptide segments 50–66 and 153–161 [21]. Leung and his colleagues cloned and expressed the TM from the new shrimp (*Metapenaeus ensis*), which had 281 amino acid residues and a molecular weight of 34 kDa [32]. Subsequently, Reese et al. extracted allergenic proteins from the brown shrimp (*Penaeus aztecus*), revealing that both the natural and recombinant proteins exhibited an α-helix conformation and were typical tropomyosin [33]. These molecules were eventually classified as TM, which was considered the major allergen in shrimp and could bind to specific IgE produced in the serum of 72% to 98% of crustacean-allergic patients. After a long period of research, it was concluded that TM is a salt-soluble protein with a molecular weight varying from 34 to 38 kDa, composed of 284 amino acids, with an isoelectric point of 4.5, and rich in glutamic acid, tyrosine, phenylalanine, arginine, and serine. It is formed by two substituents with α-helical subunits intertwined with each other to form a superhelical structure, exhibiting strong thermal stability [34,35]. It regulates muscle contraction along with actin and myosin in the organism [36]. TM from different species show a high degree of homology and structural similarity. In crustaceans such as black tiger prawn, kuruma prawn, pink shrimp, king crab, snow crab, and horsehair crab, TM is the predominant allergen, exhibiting 90% homology in amino acid sequences [37]. In addition, studies have reported that TM is also a major allergen for cockroaches and dust mites, thus TM has a certain degree of cross-reactivity.

### 3.2. AK

AK is a monomeric phosphotransferase that is widely distributed among invertebrates. It facilitates the reversible transfer of phosphoryl groups from Mg^2+^ATP to arginine, resulting in arginine phosphate and Mg^2+^ADP, and plays a crucial role in cellular energy metabolism in invertebrates [38]. Arginine kinase is available in different molecular weight forms, including a monomeric form of approximately 40 kDa, a homodimeric form of 80 kDa, and a high molecular weight form of 150–160 kDa [22]. The 40 kDa form of arginine kinase is known to be a significant allergen in shrimp [38]. One study showed that 21% of shrimp allergy patients were allergic to AK [39]. AK has been identified as an allergen in Chinese shrimp, coastal shrimp, black tiger shrimp, krill, and several other crustaceans [40]. In conclusion, AK is a glycoprotein with a molecular mass of 40–42 kDa, typically composed of 359 amino acid residues, and with an isoelectric point of approximately 6.5. Its three-dimensional structure consists of an N-terminal α-helical domain and a C-terminal α-β domain. It is stable at pH 4.0–8.0, and its IgE-binding activity is reduced at pH 9.0–11.0. However, when AK was subjected to acidic conditions (pH 1.0–3.0), its IgE-binding activity increased, suggesting that the stability and allergenicity of AK may vary under different environmental conditions. AK is stable at 30–44 °C, but as the temperature rises above 44 °C, it tends to aggregate and precipitate. The IgE-binding activity rises between 44 and 70 °C but reduces at temperatures exceeding 80 °C, which may be due to the unfolding of the AK structure at high temperatures, exposing more epitopes [41]. Research suggests that, similar to TM, AK may be a pan-allergen. A comparison of the primary sequences of AK in mollusks such as octopus, as well as crustacean seafood like shrimp and crab, indicated that their sequence homology is up to 54% [42].

### 3.3. SCP

SCP is a water-soluble EF-hand-type protein present in the muscle tissues of invertebrates and has a function in muscle relaxation [43]. The SCP has a molecular mass of approximately 20–22 kDa, contains 194 amino acids, has an isoelectric point of 4.7 and possesses four potential EF-hand calcium-binding sites, of which 2 or 3 are functional. SCP from crayfish exists as a dimer of two distinct polypeptides (α and β), which are capable of forming three isotypes: α2 (SCP-I), αβ (SCP-II), and β2 (SCP-III) [23]. It has been shown that SCP-II has weak IgE-binding activity, but sera from patients respond to all three SCP subunits, indicating that all subtypes and subunits are sensitized [44]. Morii et al. pointed out that the IgE reactivity of SCP in black tiger shrimp was primarily due to conformational IgE epitopes, some of which were stabilized by Ca^2+^ chelation, while the rest were unrelated to Ca^2+^ binding [45].

SCP has been recognized as an allergen in various shrimp species, including Penaeus monodon, Fenneropenaeus merguiensis, Procambarus clarkii, and Metapenaeus dobsonii [46]. This may be attributed to the high sequence, secondary, and spatial structural identity of SCP among crustaceans [47]. Ayuso et al. confirmed the presence of SCP as an allergen by ELISA inhibition assay and Western blotting, and the recombinant SCP was detected in a certain proportion of samples [48]. Mita et al. successfully cloned the full-length cDNA sequences of SCP from black tiger shrimp and Kuruma Shrimp, showing over 80% homology with known crustacean sequences. ELISA data demonstrated that recombinant SCP has the same IgE-binding capacity as natural SCP [49]. SCP is thermally stable and does not break down even at temperatures of up to 80 °C. The IgE-binding activity of SCP gradually increases with increasing temperature, but above 80 °C, the IgE-binding activity decreases, which may be due to changes in the protein structure and the formation of new antigenic structures caused by high temperatures [50]. SCP is highly stable under acidic or alkaline conditions: its IgE-binding activity remains unchanged under acidic (pH 1.0–5.0) and alkaline (pH 10.0–11.0) conditions [51].

### 3.4. MLC

MLC is a well-known cytoskeletal protein that regulates various processes, such as material transport, muscle contraction, and cell division [52]. MLC has been recognized as an allergen in shrimp, designated as LIT v 3.0101 [48]. MLC has a molecular weight of 18 kDa and exhibits high stability against heat, acid, and digestion [24]. Although Ayuso et al. noted in their study that the sequence similarity of MLC might be related to cross-reactivity between shrimp, cockroaches, and dust mites, it is unclear whether this is indeed the case [48]. There are two isoforms of MLC, the essential light chain (MLC1) with a molecular weight of 18 kDa and the regulatory light chain (MLC2) with a molecular weight of 20 kDa. A study demonstrated that crayfish MLC1 has four conformational epitopes and three linear epitopes, while MLC2 has one major conformational epitope and three linear epitopes [24]. Yang et al. identified three conformational epitopes of MLC1; more importantly, the key amino acids in epitope region 2 are tyrosine and phenylalanine [53]. Tyrosinase and horseradish peroxidase are commonly used in enzyme cross-linking reactions for shrimp allergens. Horseradish peroxidase primarily acts on lysine, tyrosine, phenylalanine, and cysteine residues; this provides a potential method for reducing shrimp allergy through enzyme cross-linking reactions.

### 3.5. TnC

TnC participates in calcium-dependent contraction in both skeletal and cardiac muscle and belongs to the calcium-binding protein family [54]. Although TnC was previously reported as an allergen in cockroaches and dust mites, it was not recognized as an allergen in North Sea until 2011, where it was designated as CRA C 6 [55]. Research indicated that TnC is a minor allergen in Indian black tiger prawns, American lobsters, and northern sea shrimp [25]. In Kalyanasundaram’s study, TnC was recognized as a new shrimp allergen called Pen m 6.0101 [56]. Currently, the acid–base and thermal stability properties of TnC in shrimp are not well understood. Pascal et al. confirmed that 17.2% of the 58 shrimp allergy patients were allergic to TnC [57]. Studies have shown that the EF-hand form with bound calcium exhibits higher IgE-binding activity compared to the decalcified form, suggesting that the sensitization potential of TnC may be related to its EF-hand structure. The IgE-binding epitopes and critical amino acids of allergens are crucial for further understanding allergens and clinical diagnosis; however, definitive reports on this are currently lacking.

### 3.6. HC

HC is a multifunctional protein primarily found in the hemolymph of arthropods and mollusks. It plays an important role in animal respiration and a variety of physiological activities [58]. HC is a hexamer composed of heterologous subunits with an amino acid count ranging from 630 to 660 and a molecular weight between 70 and 80 kDa. Piboonpocanun et al. extracted and purified two high-molecular-weight protein subunits (72 kDa and 75 kDa) from the giant freshwater shrimp (Macrobrachium rosenbergii) using ion-exchange chromatography. The two protein subunits showed 62.5% to 100% sequence homology with the HC sequences of other crustacean seafood species [26]. The HC from Macrobrachium rosenbergii was recognized as a novel and unique heat-resistant allergen. Additionally, HC has also been considered a major allergen in banana shrimp [59]. Subsequently, HC was shown to be an important organ-specific allergen in the hepatopancreas, but was not found in muscle, possibly due to the low level of HC in this organ, making it difficult to detect [60]. Although HC is a secondary allergen, research has revealed that the frequency of IgE binding to it is relatively high; immunoblotting techniques revealed that 38% of subjects exhibited IgE binding to TM, 38% to HC, 24% to AK, and 10% to SCP [61].

### 3.7. Triosephosphate Isomerase (TPI)

TPI is essential in the glycolytic pathway, serving at the intersection of glycolysis, lipid metabolism, gluconeogenesis, and the pentose phosphate pathways. Since TPI is widely distributed in bacteria, fungi, plants, and mammals, it may lead to complex cross-reactions between species, posing a significant threat to individuals with allergies [62,63]. Kamath et al. designated TPI as a new allergen in black tiger shrimp, characterized as a dimeric enzyme protein with a molecular weight of 26–29 kDa [27]. Subsequently, Lopez-Zavala et al. discovered that TPI in the Litopenaeus vannamei had good activity within the pH range of 7 to 9 but showed relatively low stability [64]. In recent research, a 28 kDa protein isolated from the muscle of freshwater crayfish (Procambarus clarkia) was identified as a new allergen, with serum reacting to it in 5 out of 13 patients. Native TPI contains 31.7% α-helices, 12.4% anti-parallel extension chains, and 7.4% parallel extension chains, meeting the structural criteria for α + β proteins. Its IgE-binding activity demonstrates relative stability in both the acidic and alkaline environments; however, an increase in IgE-binding activity was observed at pH 2.0 to 3.0. More importantly, as a new allergen from Procambarus clarkia, TPI shares common epitopes with filamin C, revealing potential cross-reactivity between these two allergens [28].

### 3.8. Other Allergens

Numerous additional potential allergens have been recognized in shrimp. As mentioned earlier, filamin C (FLN C) has been identified as a novel allergen in Procambarus clarkii and may cross-react with TPI. FLN C exhibits high tolerance to acids and alkalis; however, its IgE-binding activity decreases when the temperature rises to 60 °C, indicating poor thermal stability [28]. In recent research, pyruvate kinase has been recognized as a new allergen in whiteleg shrimp (Litopenaeus vannamei), exhibiting a molecular weight of 63 kDa. This allergen was found bound to specific IgE molecules in the sera of seven patients allergic to raw shrimp and four patients allergic to cooked shrimp [29]. Allergens are not only present in muscle; according to one study, the major allergen in banana shrimp is yolk proteins [59]. In addition, glyceraldehyde-3-phosphate dehydrogenase (GAPDH), enolase (EA), myosin heavy chain, and the sarcoplasmic reticulum calcium pump (SERCA) have also been reported as secondary allergens in shrimp [65]. Karnaneedi et al. identified new potential allergens through a comprehensive de novo transcriptome analysis of five shrimp species, which revealed up to 39 potential allergens not previously reported in shrimp, including heat shock proteins (HSPs), α-tubulin, trypsin, cyclosporin, β-enolase, aldehyde reductase A, and glyceraldehyde-3-phosphate dehydrogenase (G3PD); however, these have not been identified as true shrimp allergens [30]. Therefore, current research on shrimp allergens is still incomplete, and there is an urgent need to investigate potential allergens and examine their purification properties to verify their clinical sensitization, thereby establishing a comprehensive shrimp allergen system to ensure the health and safety of allergic individuals.

## 4. Effect of Processing Technologies on Shrimp Allergenicity

During processing, food undergoes a multitude of physical, chemical, and biological changes, which lead to alterations in various components, including proteins. During this process, structural changes in the allergen may lead to alterations in epitopes, resulting in the destruction or masking of existing epitopes, thereby reducing allergenicity, or the formation of new epitopes, which may increase allergenicity. Over the last few years, great emphasis has been placed on the preparation of hypoallergenic foods using processing techniques to reduce allergens. Depending on the principle, these means can be categorized into physical processing, chemical modification, and biological treatments. Table 2 summarizes the methods currently used for reducing allergenicity, their mechanisms, as well as their advantages and disadvantages.

### 4.1. Physical Method

#### 4.1.1. Thermal Treatment

Thermal processing, as a traditional food processing technique that includes steaming, boiling, pan-frying, stir-frying, and deep-frying, has the advantages of simple operation and low cost. It mainly uses high temperature to affect the properties of allergens, destroying the spatial structure and antigenic epitopes, thus reducing the immunological binding ability of allergens [11]. However, heat processing seems to have limited effectiveness in reducing allergens because most allergens in shrimp, such as TM, AK, MLC, and SCP are heat-stable. Among these, the major shrimp allergen TM is recognized for its high heat resistance. Research showed that the IgE-binding activity of TM markedly increased in boiled shrimp in comparison to raw shrimp [91]. Laly et al. [66] found that IgE activity increased by 18–27% after boiling the shrimp for 5–25 min. However, Mejrhit et al. [67] studied the sensitivity of Moroccan individuals to shrimp TM using ELISA and showed that heat treatment reduced the positive response to TM. 

Faisal et al. investigated the effect of various processing techniques (frying, boiling, freezing) on the antigenicity of TM of banana shrimp and found that frying treatment increased the antigenicity of TM by 6 to 8 times. In contrast, boiling treatment reduced the antigenicity of TM, likely due to changes in protein–solvent interactions caused by water pumping. Additionally, TM comprises a high proportion of hydrophilic amino acids like glutamic acid, lysine, arginine, and aspartic acid, which can be dissolved in water during high-temperature boiling [92]. Ozawa’s research similarly found that boiling shrimp meat chunks (about 1 g) in 10 L (*w*/*v*) for 20 min led to a TM residue of 7.1%. When the meat chunks were placed in 2000 L and treated three times at 121 °C for 20 min, the TM content was below the threshold of mandatory labeling (10 μg of protein per 1 g of food); therefore, boiling treatment is an effective method for reducing shrimp allergy [68]. In contrast, prolonged freezing treatment increased antigenicity slightly, possibly due to the formation of larger ice crystals and the disruption of hydration layers around polar residues during slow freezing, which exposes internal epitopes [92]. Studies have shown that the α-helical structure of TM (Penj1) from Japanese shrimp (*Marsupenaeus japonicus*) completely collapses at 80 °C, but it can refold back to its original state after cooling to 25 °C [93]. Although thermal processing can reduce allergens to some extent, it can also lead to nutrient loss. Additionally, high temperatures can induce a Maillard reaction between proteins and reducing sugars, which may increase allergenicity. Therefore, the effect of heat treatment is uncertain and requires more in-depth study.

#### 4.1.2. High-Pressure Processing (HPP)

HPP, as a new type of non-thermal food processing technology, mainly affects the non-covalent bonds—hydrogen, ionic, and hydrophobic bonds—between proteins, destroying the secondary and tertiary structures of the protein and causing irreversible variations to their structure, thereby reducing the allergenicity of the food [69,94,95]. Its most prominent feature is that it can better retain the original flavor and nutrients of the food.

Currently, HPP is used in the processing of various allergenic foods, including shrimp, squid, cod, oysters, and soybeans [96]. As with other heat treatments (boiling, baking, steaming, frying, microwaving), HPP results in a high reduction of IgG/IgE-binding capacity and digestive stability of shrimp [68]. Similarly, a comparative analysis of boiling, combined ultrasound and boiling, and high-pressure steaming (HPS) revealed that HPS is the most efficient approach for promoting the degradation of TM by digestive proteases and reducing the binding of TM to IgG/IgE in the simulated gastrointestinal system [69]. This is due to protein unfolding and exposure of hydrophobic residues [97]. Comparable results have been documented by Lasekan et al. [98]. In addition, the effectiveness of HPP depends on the specific conditions, and not all HPP treatments can reduce protein antigenicity. Faisal et al. revealed that HPP treatment (600 MPa) at 40 °C and 80 °C for 5 min nearly doubled the TM antigenicity of shrimp samples, which may have been because antigenic epitopes were exposed. In contrast, HPP treatment at 120 °C for 10 min decreased the number of protein bands and reduced its allergenicity by 65% [70]. Moreover, another report indicated that, compared to boiling, TM treated at 55 °C and 500 MPa for 10 min resulted in a 73.59% reduction in IgE-binding rate. In a mouse allergy model, it was found that feeding TM treated under high pressure and heat (TMH) reduced specific IgE and histamine levels in serum, and TMH exhibited almost no allergenicity [71]. Further, Liu et al. [99] demonstrated that the combined thermal/pressure process reduced protein intensity and the reduction in shrimp allergenicity was associated with the disruption of immunodominant linear epitopes (Glu177-Ser188 in tropomyosin and Gln361-Ser366 in β-actin). In addition, heated/digested stable epitopes of arginine kinase were located inside its 3D structure, preventing binding with IgE and thus maintaining hypoallergenicity. This observation indicates that high pressure has a great industrial potential in eliminating food protein allergies, e.g., high-pressure treatment (500 MPa) at 55 °C for 10 min is effective in reducing shrimp allergenicity.

#### 4.1.3. High-Intensity Ultrasound (HIU)

Ultrasonic treatment, owing to its benefits of high efficiency, energy conservation, and environmental friendliness, can significantly reduce the chemical and physical hazards brought about by traditional processing techniques, while preserving a high level of nutrients in food. It has gained attention in the field of food processing. Through the vibration of sound waves and the formation of air bubbles, strong shear forces and temperature changes can be generated in substances, which can cause alterations in the spatial structure of proteins, resulting in the destruction or concealment of their original immunogenicity sites, thus reducing their allergenicity [100,101]. HIU treatment (100–800 W, 15 min) triggered the transformation of α-helices to ss-sheet, ss-turn, and random coil in TM, resulting in the generation of protein fragments. Immune responses disappeared after HIU treatment at 800 W [102]. Similarly, ultrasonic treatment of myofibrillar protein (MP) solutions at power levels of 100 W, 300 W, and 500 W revealed that as the ultrasonic power increased, there was a significant increase in the β-sheet structure in the MP secondary structure, while the α-helix structure decreased [103]. TM treated with 30 kHz, 800 W ultrasound at 0 °C and 50 °C for 1.5 h did not show a considerable decrease in allergenicity at 0 °C, but allergenicity was significantly reduced at 50 °C, demonstrating that ultrasound attenuation of TM allergenicity requires the synergistic effect of temperature [104]. After being treated with ultrasound at 800 W, 30 kHz, and 0 °C for 30 min, the ability of cooked shrimp (*Penaeus vannamei*) to bind with IgE in the serum of allergic patients decreased by 50% [72]. Likewise, Li et al. treated TM with ultrasound at 30 Hz and 800 W for 180 min, and the finding indicated a 75% decrease in IgE-binding activity of TM, with a linear relationship between reduction rate and treatment time [73]. Ultrasonic treatment of shrimp products at 20 kHz and 400 W at room temperature revealed that allergenicity decreased from 0 to 20 min as treatment time increased. At 20 min, TM content was reduced by 76%, and the total soluble protein content decreased by 28.26% [74]. Ultrasound treatment is a highly promising technique for reducing allergens, as the presently confirmed 800 W ultrasound treatment of TM for 15 min resulted in the disappearance of its immune response. However, its effectiveness depends on ultrasound intensity, reaction time, temperature, and the characteristics of the treated substance. Further research and validation are required to fully understand and optimize this method.

#### 4.1.4. Irradiation

As a green, low-carbon, and efficient physical sensitization elimination method, irradiation technology can effectively preserve the flavor and quality of food [105]. Irradiation releases orbital electrons and forms free radicals through ionization and excitation, which react with proteins to disrupt the spatial conformation and epitope structure of allergenic proteins to reduce or eliminate allergenicity. Byun et al. [106] treated shrimp meat extracts and whole shrimp of the Pacific white shrimp (*Penaeus aztecus)* by irradiation with 60 Co-γ rays and found that irradiation could reduce the number of intact heat-stable proteins and the allergenicity of both the shrimp extract and the whole shrimp. Further, the binding capacity of TM to IgE was reduced by 81.5% after irradiation treatment at a dose of 10 kGy. The band density of the TM significantly decreased following the use of γ-irradiation to treat TM of giant freshwater prawns at doses of 10 and 15 kGy [75]. Treatment of crude extracts of shrimp with irradiation resulted in a reduction in the level of TM, and immunoblotting results revealed that the IgE activity of TM reduced as the irradiation dose increased [107]. Irradiation of TM of *Solenocera melantho* at different doses (1, 3, 5, 7, and 9 kGy) resulted in a decrease in allergenicity, with the most pronounced impact at 7 kGy, where TM’s ability to bind IgG was reduced by 59%. Electron beam irradiation led to a reduction in α-helix and β-sheet structures and an increase in β-turn structures [76]. Treatment of frozen shrimp with electron beam irradiation showed degradation of the TM, with a 20% decrease in allergenicity after 10 kGy irradiation, and a 10% increase in allergenicity after low-dose 3 kGy irradiation, which may be attributed to the fact that mild irradiation produces low concentrations of reactive oxygen species that alter the spatial structure of proteins and inadvertently increase the allergenicity of the TM protein [77]. Higher doses of irradiation can effectively reduce allergens but are more likely to affect the flavor and quality of the food. Controlling the irradiation dose is a critical step during the process of desensitization using irradiation.

#### 4.1.5. Cold Plasma (CP)

Plasma technology, as an emerging non-thermal food processing technology, has the advantages of less destruction of food components, low temperature, and shorter action time than traditional processing technologies [108]. Plasma induces changes in α-helix and β-fold contents, disrupting conformational and linear epitopes, thereby reducing allergenicity [109]. Shriver et al. [110] found that after treating the TM of white shrimp (*Litopenaeus setiferus*) with 30 kV and 60 Hz for 5 min, the allergenicity was reduced by 67%. Ekezie et al. [78] used cold argon plasma (98% argon and 2% oxygen) to treat the TM of king prawn (*Litopenaeus vannamei*) for 15 min; ELISA results indicated that the binding capacities of IgG and IgE decreased by 17.6% and 26.87%, respectively, with a transition from α-helices to β-sheets and β-turns. CP treatment alleviated allergic symptoms in mice and reduced the levels of IgG, IgE, IgG1, and IgG2a in serum, concluding that CP may prevent allergies by activating Treg cells to regulate Th1/Th2 balance [79]. Dielectric barrier discharge (DBD) cold plasma (CP, 50 kV) treatment of shrimp revealed that the molecular weight of TM increased while the protein concentration of TM reduced with prolonged treatment time. After 20 min of treatment, the α-helix content decreased by 69%, the surface hydrophobicity increased by 57.8%, and the IgE-binding capacity reduced by 96% [80]. Further studies using CP active particles confirmed that Glu131 and Arg133 in peptide P1, as well as Arg255 in peptide P2 of TM from shrimp (*Penaeus chinensis*) are IgE binding sites [111]. CP has been found to reduce allergenicity of shrimp, peanuts, and wheat, offering broad application prospects; however, it may face challenges such as high costs and potential impacts on material quality. Meanwhile, different doses and treatment times of plasma result in varying degrees of allergen reduction. It has been confirmed that treating food with 50 kV DBDCP for 20 min can reduce the IgE-binding ability of TM by 96%, showing a relatively good reduction effect.

#### 4.1.6. Other Physical Methods

Microwaves may have significant effects on activity and structural properties of proteins and peptides [112]. Microwave processing (2.45 GHz, 1000 W, 75–125 °C, 5–15 min) decreased the intensity of TM bands with increasing processing temperature and time. The allergenicity of TM reduced by 75% after microwave treatment at 125 °C for 15 min [113]. Pulsed light can also alter the allergenicity of TM. Treatment with pulsed light (3 pulses/s, 10 cm from the light source) showed a reduction in the grayscale of TM in SDS-PAGE after 4 min, and at 4–6 min of treatment, IgE activity was significantly decreased. The combination of boiling and pulsed-light treatment resulted in the greatest reduction in the allergenicity of TM [114].

**Figure 1:**
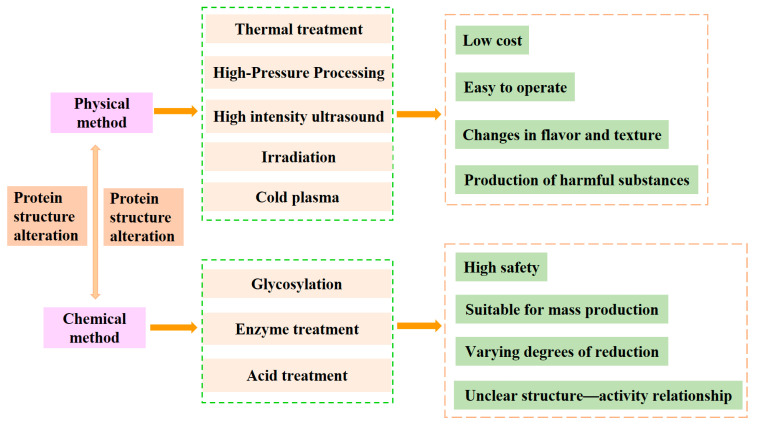
Processing techniques for allergen reduction and their advantages and disadvantages.

Physical processing methods are considered safe as they do not involve chemical reagents, and the use of integrated equipment makes them more suitable for large-scale production. However, since the mechanism of the interaction between various physical fields and allergens has not yet been clarified, the structure–activity relationship of “exposure conditions—conformational changes—sensitization” during the processing is not well understood, and the trends of changes in the antigenicity of the products after the technical treatment vary; thus, further in-depth studies are needed (Figure 1).

### 4.2. Chemical Method

#### 4.2.1. Glycosylation

Glycosylation of proteins in food refers to the non-enzymatic chemical reaction between amino compounds in proteins (primarily lysine with ε-amino groups) and carbonyl compounds (mainly reducing sugars). It is also known as the Maillard reaction and primarily takes place in foods that have undergone heat treatment and long-term storage. The chemical process of the Maillard reaction is extremely complex, involving a sequence of chemical rearrangements, including condensation, oxidation, and hydration. The initial products of the reaction are Schiff bases, which subsequently undergo Amadori rearrangement and further oxidative modifications (glycoxidations), ultimately forming advanced glycation end products (AGEs). These AGEs, as neoantigens, may trigger new immune responses. For example, Nε-(carboxymethyl) lysine (CML), one of the AGEs, can act as an immune epitope to enhance allergic reactions [115]. The receptor for AGEs (RAGE), a member of the immunoglobulin superfamily, is widely present on the surface of a wide range of allergy-associated immune cells. Studies have shown that Ara h 1 and Ara h 3 modified with CML can be preferentially recognized by RAGE on cell membranes, leading to the activation of RAGE receptors, thereby promoting allergic responses [116]. Elements like the number and type of reducing sugars can affect the effectiveness of glycosylation treatments in reducing allergenicity. It was shown that galacto-oligosaccharide-glycosylated TM and maltopentaose-glycosylated TM were hypoallergenic compared to TM, resulting in milder allergic symptoms in mice. On the other hand, fructo-oligosaccharide-glycated TM did not have a notable impact on allergenicity due to the production of new allergens associated with AGEs, which may offset the loss of epitopes induced by the glycosylation [117]. The Maillard reaction (ribose, arabinose, galactose, glucose, and maltose) can influence the allergenicity of TM and AK, with galactose, glucose, and arabinose reducing the allergenicity of TM, and arabinose decreasing AK allergenicity [118]. The Maillard reaction (ribose, galacto-oligosaccharides, chitosan-oligosaccharides) shifts the TM conformation from α-helix to β-sheet, resulting in a 60% reduction in allergenicity. Additionally, there is a certain correlation between the degree of protein grafting, secondary structure content, and the IgE-binding capacity of the product [81]. Lyu et al. [82] found that 4000 mmol/L ribose significantly reduced the IgE-binding ability of TM and inhibited the release of cytokines from RBL-2H3 cells. At the same time, the spatial structure of TM protein underwent significant changes, mainly due to the modification of the phenylalanine, isoleucine, and methionine residues by ribose, altering the antigenic epitopes of the TM protein. Glycosylation of galacto-oligosaccharides (TM-GOS), mannan-oligosaccharides (TM-MOS), and maltopentaose (TM-MPS) reduced the allergenicity of TM, whereas glycosylation of TM-FOS increased the allergenicity of TM, with α-helix content decreasing from 78.7% to 60.7% (TM-GOS), 66.7% (TM-FOS), 71.3% (TM-MOS) and 68.9% (TM-MPS) [116]. TM glycosylated with glucose (TM-G) resulted in weaker allergic reactions in mice and mast cells, which could be attributed to the fact that glucose disrupts the epitope with the glycation site [119]. Yuan et al. [120] found that after glycosylation catalyzed by transglutaminase, the α-helix content of TM protein increased and the free amino group content decreased, resulting in reduced IgG/IgE-binding ability. Overall, glycosylation can serve as an effective method for reducing shrimp allergen sensitivity. However, the complexity of the reaction processes, numerous influencing factors, and the potential formation of AGEs make it difficult to control. Further studies on the effects of different sugars on allergens are needed to develop more effective methods for reducing shrimp allergenicity.

#### 4.2.2. Enzyme Treatment

Enzyme treatment is regarded as an effective approach for mitigating the allergenicity of shrimp allergens. It typically involves two processes: The first is enzymatic hydrolysis. Proteases hydrolyze and break the peptide bonds of allergens, disrupting their spatial structures and linear epitopes, thus achieving the goal of reducing the allergenicity of allergens [121]; the other is enzymatic cross-linking, which refers to intramolecular or intermolecular cross-linking reactions induced by enzymes that alter the molecular weight of proteins, inducing protein aggregation and disrupting secondary structures and IgG/IgE-binding epitopes. The enzymatic hydrolysis process is efficient, safe, and environmentally friendly. Studies have confirmed the potential of pepsin, trypsin, α-chymotrypsin, and protease P to degrade allergens [122]. Immunoblot analysis revealed that chymotrypsin hydrolysis at both 37 °C and 50 °C effectively inhibited the immunoreactivity of TM compared to trypsin hydrolysis at 37 °C [123]. Liu et al. [83] treated grass prawn with pepsin, trypsin, and chymotrypsin and found that the TM exhibited a degree of resistance to pepsin digestion and was completely digested by trypsin and chymotrypsin. Mejrhit et al. [67] found that when TM was treated with pepsin (30 μg/mL) at pH 2 for 2 h, the recognition rate of TM by serum IgE from 20 patients decreased by an average of 89.4%.

Using laccase or laccase/caffeic acid to crosslink TM, experiments revealed a decrease in IgG/IgE-binding activity, an increase in gastrointestinal digestibility, and lower degranulation levels in RBL-2H3 and KU812 cells [124]. Tyrosinase and horseradish peroxidase cross-linking of TM resulted in 34.5% and 63.5% reductions in IgE-binding capacity, respectively, as well as an increase in oral tolerance in mice [125]. Investigations demonstrated that the treatment of TM with transglutaminase (TG) and tyrosinase (Tyr) decreased IgE-binding capacity, with the proportion of α-helices decreased by 20.1% and 15.2%, while β-turns increased by 5.8% and 6.2%, respectively, which indicated that enzymatic cross-linking induced a transition from α-helices to β-turns, altering the protein structure and reducing allergenicity [84]. Similarly, Fu et al. confirmed that enzyme cross-linking led to the formation of additional β-turns and the exposure of tryptophan, tyrosine, and phenylalanine residues within the molecule, disrupting the conformation of the TM [126]. Additionally, TM treated with tyrosinase (Tyr) and caffeic acid (CA) suppressed levels of IgG1, IgE, histamine, and mast cell protease-1 (mMCP-1) in mouse serum, and may alleviate allergic reactions by modulating the Th1/Th2 balance [127]. The greatest reduction in the capacity of TM binding to IgG (37.19%) and IgE (49.41%) was also reported under the treatment of 2000 nkat/g Tyr +CA [128]. Enzyme-catalyzed protein cross-linking and hydrolysis are mild and effective methods for reducing the allergenicity of shrimp allergens.

#### 4.2.3. Acid Treatment

The changes in acidity or alkalinity in the food matrix environment are common during processing, and “alkaline dissolution and acid precipitation” is one of the basic principles for producing isolated proteins in the current industrialized production. Currently, there are few reports on the changes in allergenicity of shrimp products after acid treatment. Eden et al. [85] found that shrimp extract, which had been cooked and pre-soaked in vinegar for 8 h, exhibited lower allergenicity. Lasekan et al. [86] found that, compared with shrimp marinated at pH 4.8 and a control group, the immunoglobulin E (IgE)-binding capacity of TM in the soluble protein fraction of shrimp marinated at pH 1.0–3.5 was significantly lower. The structural modification of allergens by acid treatment, and consequently the reduction of their sensitizing properties, was mainly achieved by internal amino acid protonation of the proteins. Under conditions below the isoelectric point of the protein, the excess H^+^ in solution will protonate the free amino and carboxyl groups in the protein molecules, and mutual repulsion of the same charge will occur within the molecule, leading to the aggregation of the protein, which in turn destroys its conformational epitopes and leads to a reduction in sensitization. However, in the presence of large numbers of linear epitopes, acidic conditions may not be able to reduce the sensitization [129].

#### 4.2.4. Other Chemical Treatments

In addition to the common glycosylation and enzymatic methods used to reduce the allergenicity of TM proteins, other chemical treatments such as acrylamide, 2,2′-azobis(2-amidinopropane) dihydrochloride, malondialdehyde, polyphenols, etc., can also be employed. At 25 °C, incubating TM of *Penaeus vannamei* in 1 mmol/L of acrolein for 24 h resulted in significant reductions in TM-specific IgE and IgG1 levels and histamine content, and the IgE-binding capacity of TM diminished as the concentrations of acrolein increased [130]. Similarly, Lv et al. demonstrated that with increasing concentrations of acrolein, the binding capacity of IgE decreased, the secondary structure was denatured, and acrolein altered the free amines of lysine, tyrosine, and histidine residues [131]. Modification of TM with 2,2′-azobis(2-amidinopropane) dihydrochloride led to a significant decrease in TM protein-specific IgE/IgG1, histamine, and mMCP-1 levels, and new bands appeared in the TM, indicating cross-linking [132]. The addition of linoleic acid (LA) significantly reduced IgG/IgE immunoreactivity, digestibility, and immunodetection rate of TM. LA binding to TM exposes more buried hydrophobic residues, and the three-dimensional structure of TM undergoes a major change, with an increase in particle size and hydrophobic surface area [133].

Malondialdehyde-induced cross-linking of TM resulted in a decrease in α-helix content while simultaneously increasing the contents of β-sheet, β-turn, and random coil contents [87,88]. TM of shrimp treated with different concentrations of malondialdehyde exhibited slight degradation, improving pepsin digestion stability; however, the TM-IgE binding capacity showed a slight decrease after gastric digestion and a significant reduction following intestinal digestion [134].

Over the past few years, reports have indicated the formation of low-allergenicity proteins through protein–small molecule interactions, primarily focusing on the interaction between allergens and polyphenols. The principle is to utilize non-covalent intermolecular forces, such as hydrogen bonds, van der Waals forces, and hydrophobic interactions, to alter the conformational structure of allergens in the system, thereby affecting their antigenicity [135]. Polyphenols induce alterations in the molecular weight of TM while also reducing the IgG/IgE-binding capacity of TM and reducing the sensitization of TM in mouse models [136]. Conjugation of TM with *Sargassum fusiforme* polyphenol (SFP) led to conformational instability, remarkably reducing the binding capacities of IgG and IgE, weakening the TM-stimulated anaphylactic responses in mast cells, and exhibiting in vivo anti-allergic properties in BALB/c mouse models [137]. The covalent binding of TM with quercetin and chlorogenic acid altered the allergenic epitopes of shrimp TM, reducing the levels of IgE, IgG, IgG1, histamine, and mMCP-1 in serum, thereby decreasing its potential allergenicity [138].

Chemical methods have the advantages of easy operation, low cost, and high efficiency, but enzymatic hydrolysis is prone to loss of protein processing function, affecting the sensory qualities and texture of food. Glycosylation and cross-linking reactions are difficult to control and have the risk of generating hazardous substances (e.g., AGEs) (Figure 1).

### 4.3. Biological Method

The fermentation method mainly uses microorganisms to decompose or denature allergenic proteins, destroying their spatial conformations and antigenic epitopes to reduce allergenicity [139]. Fermentation can break down macromolecules into small molecules, improving the texture, flavor, and nutritional value. Compared to shrimp, the specific IgE response to Indonesian commercial Terasi (ICT, fermented shrimp paste) was significantly reduced, but all ICT samples still possessed IgE-binding ability [140]. Further addition of fermentation strains accelerated the fermentation of the raw materials, effectively promoting the degradation of shrimp proteins and reducing the IgE-binding capacity of TM [141]. Park et al. [142] found that fermentation could reduce the binding capacity of TM in shrimp to the serum of allergic individuals. And when the fermentation temperature was 25 °C, the allergenicity of TM started to decrease after 12 d of fermentation, and lowering the fermentation temperature delayed the onset of the reduction in the allergenicity of TM. Fu et al. [89] found that oral administration of *Lactobacillus casei* could alleviate allergic symptoms and intestinal epithelial damage in BALB/c mice induced by shrimp TM. Furthermore, fermentation with *Lactobacillus helveticus* TS6024 and *Lactobacillus acidophilus* 6005 reduced shrimp allergenicity by 78.97% and 70.09%, respectively, with a marked decrease in the band intensity of TM (36 kDa) [90].

### 4.4. Synergistic Reduction Techniques

Most shrimp allergens are resistant to acid, alkaline, high temperatures, and digestion, making traditional processing methods ineffective at reducing their allergenicity. Prolonged high temperature treatments can notably decrease the allergenicity of shrimp allergens, but will negatively affect the quality of shrimp meat. Currently, some non-thermal processing methods, such as high pressure, ultrasound, plasma, and irradiation, have shown preliminary results on abating the allergenicity of shrimp allergens; however, the effectiveness of these methods varies, and they can also affect the quality of the shrimp meat. Therefore, some scholars have adopted combined methods to reduce allergenicity.

During thermal processing, Methylglyoxal (MGO) can cause protein bands to migrate. MGO modifies the Lysine, Arginine, Aspartic acid, and Glutamine residues of tropomyosin (TM) during thermal processing, thus disrupting and/or masking TM epitopes. In vivo, TM-MGO significantly reduced the levels of antibodies, histamine, and mast cell protease 1 in the serum [143]. Mice fed shrimp processed by steaming (100 °C, 5 min) followed by reverse pressure sterilization (0.15 MPa at 110 °C for 20 min) showed significantly lower levels of specific IgE and IgG1, as well as degranulation reactions, vascular permeability, and allergic symptoms compared to mice fed raw or steamed shrimp [99]. Grilled and pressure-cooked shrimp significantly reduced specific antibodies and histopathological morphology in mice. This is because counterpressure sterilisation leads to protein aggregation, which hides the heat/digestion-stable epitopes of AK (Glu59-Ser63, Asn112-Lys118, Leu131-Phe136) [144]. The combination of the Maillard reaction (shrimp meat with galactose) and high-pressure processing at 115 °C significantly altered the macro-structure of shrimp meat, enhanced its digestibility, and markedly reduced the IgG/IgE-binding activity. The reduction in IgE-binding activity is likely attributed to the modification of lysine, arginine, and cysteine residues in the antigenic epitopes [145]. Cold plasma (DBD, 60 kV, 1.0 A) combined with glycation treatment (4 h, 80 °C) reduced the IgE-binding ability of TM by as much as 40%, whereas single treatment with either cold plasma or glycation reduced it by less than 5% [146].

## 5. Conclusions and Future Perspectives

In the past few years, remarkable progress has been made in the study of shrimp allergens and allergen reduction techniques. Different processing methods can lead to a series of changes in allergens, including peptide bond hydrolysis, non-covalent bond aggregation, protein denaturation, glycosylation, cross-linking, and protein fragment hydrolysis. The decrease in antigenicity may be related to changes in α-helix, β-fold, and random coil structures, which are generally achieved by inducing the transformation of α-helix into other structures. In addition, allergenicity is typically assessed by indicators such as IgE-binding capacity, protein band intensity, in vitro protein digestibility, surface hydrophobicity, and histamine levels. In addition, shrimp allergens such as TM can cross-react with other invertebrate (e.g., insect) allergens, and in light of the increasing introduction of novel food products such as insect power into the global diet, the effects of processing techniques on shrimp allergens in this paper could provide references for the development of novel food products. Although the relevant research has achieved remarkable achievements, there are still several issues that need to be addressed: (1) Allergen identification: While some major or minor shrimp allergens have been identified, there is still a need to explore potential allergens in shrimp and establish a comprehensive shrimp allergen system to achieve better allergy diagnosis and prevention. (2) Standardized experimental conditions: Food processing research benefits from highly standardized experimental conditions and methods. Allergen reduction methods (e.g., heat treatment, high pressure, ultrasound, and irradiation) rely on the choice of experimental conditions. However, current investigations in this field are insufficient, and there is a lack of precise reference standards. (3) Quality assurance: Current desensitization and abatement technologies primarily focus on allergenicity reduction, with less consideration given to the nutritional value, quality, texture, and economic value of food. How to balance nutrition and safety is an important research topic. (4) Synergistic technologies: Synergistic technologies can not only improve the abatement effect but also ensure the physicochemical properties of the food. However, compared to single technologies, synergistic technologies require more parameters to be controlled, and their effects may be either synergistic or antagonistic; therefore, it is necessary to explore more possibilities and conduct more diversified research. (5) Universality: Current research mainly focuses on the abatement of purified allergens, while fewer studies have been conducted on whole shrimp, shrimp meat, or shrimp paste. Whether similar reduction effects can be achieved in these forms requires further confirmation.

## Figures and Tables

**Table 1:** List of identified and characterized shrimp allergens [8].

Biochemical Names	MW (kDa)	Function	Source	Heat Resistance	References
Tropomyosin	34–38 kDa	Regulates muscle contraction	*Penaeus aztecus* (Pen a l) *Penaeus indiana* (Pen I 1)	Yes	[20,21]
Arginine kinase	40 kDa	Cellular energy metabolism	*Penaeus monodon* (Pen m 2) *Litopenaeus vannamei* (Lit v 2)	No	[22]
Sarcoplasmic calcium-binding protein	20–22 kDa	Regulates muscle relaxation	*Penaeus monodon* (Pen m 4) *Litopenaeus vannamei* (Lit v 4)	Yes	[23]
Myosin light chain	18–20 kDa	Material transport, muscle contraction, and cell division	*Procambarus clarkii*	Yes	[24]
Troponin C	18 kDa	Calcium-dependent contraction in both skeletal and cardiac muscle	*North Sea Shrimp* (Cra c 6)	no data	[25]
Hemocyanin	60–80 kDa	Animal respiration and physiological activities	*Macrobrachium rosenbergii* (Mac ro 2)	Yes	[26]
Triosephosphate isomerase	26–29 kDa	Engage in glycolysis, lipid metabolism, gluconeogenesis	*black tiger prawn*	No	[27]
Filamin C	90 kDa	Stabilizes the cytoskeleton	*Procambarus clarkii*	No	[28]
pyruvate kinase	63 kDa	Catalytic enzyme	*Litopenaeus vannamei*	no data	[29]
Glyceraldehyde-3-phosphate dehydrogenase	37 kDa	Catalytic enzyme	*Fenneropenaeus merguiensis*	no data	[30]
Enolase (EA)	50 kDa	Enzyme protein	*Melicertus latisulcatus* *Fenneropenaeus merguiensis*	no data	[30]
Endoplasmic reticulum Ca^2+^ A Tpase	113 kDa	Enzyme protein	*Melicertus latisulcatus* *Fenneropenaeus merguiensis*	no data	[30]
Myosin heavy chain	18–20 kDa	Provide energy, muscle contraction	*Melicertus latisulcatus* *Fenneropenaeus merguiensis*	no data	[30]

**Table 2:** Effects of mitigation technologies on shrimp allergens.

Processing Method	Mechanism	Effect on Allergenicity	Advantages	Limitations	References
Thermal treatment	Hydrolysis, aggregation, and folding of allergenic proteins Damage the secondary and tertiary structures of allergen proteins	Increased IgE-binding capacity increased by 18–27% Reduced	The operation is simple No chemicals are used and it is harmless to the human body	The degradation of allergens is limited Heat-sensitive nutrients decompose easily It is possible to generate new epitopes or expose existing ones	[66,67,68]
High-pressure processing	Affects covalent bonds, such as hydrogen, ionic, and hydrophobic bonds Causes reversible or irreversible structural modifications in proteins Leads to denaturation and aggregation of allergens	Reduced IgG/IgE-binding capacity Reduced allergenicity by 65% IgE-binding capacity reduced by 73.59%	Food flavor and natural substances are not affected Significantly reduce allergenicity	High voltage equipment is required	[69,70,71]
High-intensity ultrasound	Modify food proteins by inducing mechanical, physical, and chemical/biochemical changes through the cavitation phenomenon	IgE-binding capacity reduced by 50% IgE-binding capacity reduced by 75% Reduced by 76%	Substantially reduce the allergenicity of allergens Preserve the nutrition and flavor of food to a greater extent	Specialized equipment is required The technology is not mature and needs further optimization	[72,73,74]
Irradiation	Leads to denaturation, aggregation, hydrophobicity, and structural modification of proteins	Reduced band density of the TM IgG-binding capacity reduced by 59% Allergenicity reduced by 20%	Show enough potential for mitigating shrimp allergenicity No toxicological and microbiological hazard	Depends on the choice of irradiation dose It is hard for the public to accept The equipment is expensive and the operation is complex	[75,76,77]
Cold plasma	Disrupt protein structure, cause changes to α-helix and β-fold contents	Allergenicity reduced by 67% IgE-binding capacity reduced by 26.87% IgE-binding capacity reduced by 96%	Significantly reduce allergenicity Low energy consumption, low temperature, and short time consumption	Specialized equipment is required Possible induction of lipid oxidation	[78,79,80]
Glycosylation	AGEs produced by oxidation act as new allergens The linear epitope and the conceived epitope are broken	Allergenicity reduced by 60% Reduced IgE-binding capacity	Significantly reduce allergenicity Enhance the color and flavor	It leads to the loss of nutrients It causes formation of harmful substances and anti-nutrients	[81,82]
Enzyme treatment	Breakdown of allergens Proteins are modified by enzyme cross-linking, changing the structural properties of the protein	IgE-binding capacity reduced by 63.5% IgE-binding capacity reduced by 49.41%	The process is simple and is carried out under mild conditions. No toxic chemicals are introduced High efficacy in reducing IgE-binding capacity	Difficulty controlling the reaction, the reaction takes a long time May produce bitter peptides, which can affect the taste of the product	[83,84]
Acid treatment	Induce protein denaturation by altering the conformational structure	Exhibited lower allergenicity Reduced the IgE-binding capacity	Show reduction in the IgE-binding capacity	Decrease the solubility of allergenic protein in extraction solution	[85,86]
Malondialdehyde crosslinking	Alter the secondary structure of proteins	Reduced TM–IgE-binding capacity	Show enough potential for mitigating shrimp allergenicity	The added chemicals are harmful to the quality of food It is difficult to apply in industrial production	[87,88]
Biological method	Decompose proteins Alter the spatial conformation of allergens and destroy antigenic epitopes	Reduced Reduced by 78.97%	Break down proteins and promote absorption in the body Add flavor to food	Difficulty controlling the reaction	[89,90]

## Data Availability

No new data were created or analyzed in this study. Data sharing is not applicable to this article.
